# Cord Blood Lin^−^CD45^−^ Embryonic-Like Stem Cells Are a Heterogeneous Population That Lack Self-Renewal Capacity

**DOI:** 10.1371/journal.pone.0067968

**Published:** 2013-06-28

**Authors:** Cesar Alvarez-Gonzalez, Richard Duggleby, Barbora Vagaska, Sergio Querol, Susana G. Gomez, Patrizia Ferretti, Alejandro Madrigal

**Affiliations:** 1 Anthony Nolan Research Institute, London, United Kingdom; 2 Cancer Institute, University College London, London, United Kingdom; 3 Development Biology Unit, Institute of Child Health, University College London, London, United Kingdom; 4 Anthony Nolan Cell Therapy Centre, Nottingham, United Kingdom; 5 Banc de Sang i Teixits, Barcelona, Spain; French Blood Institute, France

## Abstract

Human umbilical cord blood (hUCB) has been proposed to contain not only haematopoietic stem cells, but also a rare pluripotent embryonic-like stem cell (ELSc) population that is negative for hematopoietic markers (Lin^−^CD45^−^) and expresses markers typical of pluripotent cells. The aim of this work was to isolate, characterise and expand this ELSc fraction from hUCB, as it may provide a valuable cell source for regenerative medicine applications. We found that we could indeed isolate a Lin^−^CD45^−^ population of small cells (3–10 µm diameter) with a high nucleus to cytoplasm ratio that expressed the stem cell markers CD34 and CXCR4. However, in contrast to some previous reports, this fraction was not positive for CD133. Furthermore, although these cells expressed transcripts typical of pluripotent cells, such as *SOX2*, *OCT3/4*, and *NANOG*, they were not able to proliferate in any of the culture media known to support stem cell growth that we tested. Further analysis of the Lin^−^CD45^−^ population by flow cytometry showed the presence of a Lin^−^CD45^−^Nestin^+^ population that were also positive for CD34 (20%) but negative for CXCR4. These data suggest that the Lin^−^CD45^−^ stem cell fraction present in the cord blood represents a small heterogeneous population with phenotypic characteristics of stem cells, including a Lin^−^CD45^−^Nestin^+^ population not previously described. This study also suggests that heterogeneity within the Lin^−^CD45^−^ cell fraction is the likely explanation for differences in the hUCB cell populations described by different groups that were isolated using different methods. These populations have been widely called “embryonic-like stem cell” on the basis of their phenotypical similarity to embryonic stem cells. However, the fact they do not seem to be able to self-renew casts some doubt on their identity, and warns against defining them as “embryonic-like stem cell” at this stage.

## Introduction

Human umbilical cord blood stem cells (HUCBSC) are potentially a very important source of stem cells for tissue repair, as their use would circumvent the ethical issues involved with embryonic stem cells. Furthermore, HUCBSC have already been extensively used for treating many haematological related disorders, hence they are believed to be safe [1], [2]. Whereas there are a number of reports suggesting the human umbilical cord blood (hUCB) can give raise to different lineages, the issue of their origin and of the existence of a truly pluripotent population with the hUCB has been a matter of debate. Recently, much work has focused on the characterization of HUCBSC and their potential to differentiate into different lineages, including neural cell types [1]. From the studies aimed at characterizing population(s) of putative stem cells present in the hUCB, the population of stem cells lacking expression of CD45/leukocyte common antigen (LCA) is of particular interest [3], [4], [5].

Relatively little information on this CD45 negative (CD45^−^) population is currently available. It has been reported to also lack expression of mature lineage (Lin) markers (CD2, CD3, CD14, CD16, CD19, and CD56), but to express several stem cell markers, such as CD34, CD133, and CXCR4. In addition, it appears to express transcripts typical of pluripotent stem cells, such as *SOX2*, *OCT3*/4, and *NANOG* [4]. CD45^−^ populations have been isolated from bone marrow and hUCB using a range of different protocols, characterized using different parameters and the resulting cells have been assigned several different names [6], [7], [8], [9], [10], [11], [12]. These include: very small embryonic-like stem cells (VSELs) [6], cord-blood-derived embryonic-like stem cells (CBEs) [7], multipotent adult progenitor cells (MAPC) [8], serum deprivation-induced bone marrow stem cells (SD-BMSC) [9], marrow-isolated adult multi-lineage inducible (MIAMI) cells [10], multipotent adult stem cells [11], and unrestricted somatic stem cells (USSCs) [13]. While some of the features of the CD45^−^ population in hUCB reported by the different groups are the same, others differ, and the properties, function, and plasticity of this cell population remain unclear.

The aim of this study was to further characterize the Lin^−^CD45^−^ population(s) present in the hUCB to clarify the discrepancy in the features of this population reported in the literature and assess their behaviour. The Lin^−^CD45^−^ population was isolated using a magnetic cell isolation method modified from McGuckin et al. [3] from the cord blood nucleated cell fraction purified either by red blood cell lysis or by density gradient centrifugation. This excluded most of hematopoietic population including the Haematopoietic Stem Cells (HSC). Analysis of the Lin^−^CD45^−^ population demonstrated the presence of different subpopulations both in regard to cell size and stem cell marker expression. Furthermore, although we could detect expression of markers of pluripotency, such *SOX2*, *OCT3*/4, and *NANOG*, no proliferation of these cells occurred under any of the culture conditions tested. Therefore, the embryonic stem cell-like molecular phenotype is not mirrored by self-renewing capability. Altogether, our study does not support a significant presence of embryonic stem cell-like cells in the hUCB, and suggests the presence of a heterogeneous population within the Lin^−^CD45^−^ cell fraction that can at least partly reconcile differences reported in different studies.

## Materials and Methods

### Cell Sources

Whole cord blood from non-clinical standard units was supplied by the Anthony Nolan Cell Therapy Centre (http://www.anthonynolan.org/Healthcare-professionals/Cord-blood-services.aspx) from consented mothers with healthy full-term pregnancies.

Total nucleated cells (TNCs) were prepared using two different protocols. The first protocol involved lysis of red blood cells using BD Pharm Lyse (Cat: 555899). Briefly, hUCB was centrifuged at 1000g to remove the cord plasma as reported by Bhartiya, D., et al. [5]. The pellet containing red blood cells (RBCs) and TNCs was treated with lysing buffer (dilution 1∶5) for 15 minutes at room temperature (RT), in accordance with manufacturer instructions, and centrifuged at 1000g for 10 minutes at RT (for detailed protocol, see Methods S1). Cells were washed twice with phosphate buffer saline (PBS, AccuGENE® Lonza Cat: 51226).

The second protocol involved the use of density gradient centrifugation over Ficoll-PaqueTM PREMIUM (GE healthcare). Cord blood mononuclear Cells (CBMCs) were isolated from the interphase after gradient centrifugation (at 840g) over Ficoll-PaqueTM PREMIUM and treated once with lysis buffer due to residual RBCs. The pellet was washed twice with PBS, resuspended, and centrifuged at 1000g to isolate the light-density mononuclear cells.

A Human Neural Stem Cell (hNSC) line that was generated and expanded [K. Pong, Nicholas, A. P., Subramanian, V., Thompson, P. R., Ferretti, P. Peptidylarginine deiminase 3 - a novel early mediator of calcium-induced death in human neural stem cells. Unpublished data, submitted, 2013] as previously described [14] was used as a positive control for Nestin, CD133, and Sox2.

A line of Mesenchymal Stem Cells (UC-MSC) from Wharton’s jelly generated as described previously by Weiss et al [15] was used in some experiments as a control for gene expression analysis.

### Flow Cytometry

Flow cytometry analysis was carried out with a BD FACSCalibur TM. Data analysis was performed using FlowJo 6.4.7 software (Tree Start, Inc. 1997–2006). The monoclonal antibodies used were: from BD Bioscience: Lineage Cocktail 1 (FITC; Cat: 340546), CD34 (APC; clone: 581), CXCR4 (also known as CD184 Cat: 555974, clone: 12G5), 7AAD (Cat: 51-68981E), CD41a (FITC and APC; clone: HIP8), Oct3/4 (PerCP-Cy TM 5.5; clone: 40/Oct-3), Anti-Sox2 (Alexa Flour 488; clone: 245610), SSEA-4 (FITC and PE; clone:MC813-70), and Nestin (PE; clone: 25NESTIN); from eBioscience, CD45 (biotin; clone:HI30), CD235a (biotin; clone: HIR2), Hematopoietic Lineage (FITC including CD2, CD3, CD14, CD16, CD19, CD56, and CD235a; Cat: 22-7778); from Miltenyi Biotec: CD45 (FITC, PE, PerCP, and APC clone: 5B1); CD133 (PE and APC clone: 293C3), and anti-biotin antibodies PE and APC (Bio3-18E7). For cell surface labelling, cells were incubated with antibodies diluted in FACS buffer (2.5% FBS in PBS 1x) for 10–15 minutes at 4°C (with the exception of CXCR4 where the incubation was for 30 minutes) and then washed twice with FACS buffer for 3–4 min. For intracellular staining, cells were fixed with 4% paraformaldehyde for 20 minutes at 4°C, permeabilized with Perm/Wash Buffer I (BD, Cat: 557885) for 5 minutes at RT, and stained with SOX2, OCT3/4, or Nestin for 30 minutes and washed twice for 3–4 minutes with FACS buffer. For negative controls cells were stained using FACS buffer only.

### Magnetic Cell Isolation

After removing the erythrocytes using either the lysis buffer or the gradient centrifugation method, TNCs were centrifuged at 1000g for 10 minutes the pellet resuspended in MACS buffer (PBS 1x, 2mM EDTA, and 1% BSA) at 4°C, and cells incubated for 10 minutes at 4°C in the dark with the following biotin-conjugated antibodies: from eBioscience: CD235a (HIR2), CD11b (ICRF44), CD123 (6H6), CD45 (HI30); from AbD Serotec: CD61 (PM6/13). Anti-Biotin MicroBead-conjugated antibodies (Miltenyi Biotec, Cat: 130-090-485) were then added and incubated for 15 minutes at 4°C in the dark. Finally the cells were passed through LD magnetic columns (Miltenyi Biotec, Cat: 130-042-901) according to the manufacturer instructions and the negative fraction (-F) collected.

### RT-PCR and qPRC

Three cords were pooled for magnetic cell isolation. Total RNA was isolated from the cell pellet using RNeasy Mini Kit (Qiagen, Cat: 74104) according to manufacturer’s instructions. cDNA was prepared using D6N random hexamer (Applied Biosystem) annealed at 80°C for 10 minutes followed by reverse transcription using MMLV-Ez (200 U/µl) (Promega), MLV-RT buffer (5X) (Promega), dNTP (0.2 mM) (Bioline), RNasin Ribonuclease Inhibitor (2500 U/µl) (Promega) and RNase free water. cDNA was amplified in a Veriti thermal cycler (Applied Biosystems, Foster City, CA) with GoTaq (Promega) using primers and conditions previously described by Guasti et al. [16].

Real-time quantitative polymerase chain reaction (qPCR) was performed with an ABI Prism 7500 sequence detection system (Applied Biosystems) and the QuantiTect SYBR Green PCR Kit (Qiagen) according to the manufacturer’s instructions. PCR reactions were set up in triplicates in 96 well plates. The housekeeping gene GAPDH was used as an internal control to normalize expression levels and data were analysed using the 2 −ΔΔCT method.

### Cell Culture

For colony-forming unit (CFU) assessment, all cells recovered from –F were plated in Methylcellulose medium supplemented with recombinant cytokines as previously described [17] and haematopoietic colonies scored after 14 days. One million of TNCs (containing around of 1000–2000 HSC) from positive fraction (+F) were plated as a control.

Absolute counts of both Lin^−^CD45^−^CD34^+^ and Lin^−^CD45dimCD34^+^ cells were performed as described previously [17] in the –F fraction to estimate the number of HSC contaminants plated.

For cell survival and growth studies cells were plated either on Matrigel (BD Matrigel TM hESC-qualified Matrix, Cat: 354277) or laminin (SIGMA, Cat: L2020 added to the medium conditions). Five different culture media (A–E) were used, and their composition is shown in Table 1. The components were DMEM/F-12 (1∶1) (GIBCO, Cat: 31330), RHB-A® (StemCells Inc. Cat: SCS-SF-NB-01), StemSpan® SFEM (STEMCELL TECHNOLOGIES, Cat:09600) MethoCult® GF H84434 (STEMCELL TECHNOLOGIES); B27 and N2 neuronal supplements (PAA, Cat: F01-005 and F005-004, respectively); FGF-2 (PeproTech, Cat: 100–118B; or Miltenyi Biotec Cat: 130-093-838), EGF (PeproTech, Cat: AF-100-15), Stem cell Factor (Miltenyi Biotec, Cat: 130-093-991), Flt3-Ligand (Miltenyi Biotec, Cat: 130-093-854). The medium was changed every 3–4 days and cells monitored for up to one month.

**Table 1:** Stem cell culture conditions used to expand the Lin^−^CD45^−^ stem cells.

	A	B	C	D	E
Medium	DMEM/F-12	DMEM/F-12	DMEM/F-12	RHB-A®	StemSpan® SFEM
FBS (10%)		✓			
Supplements					
FGF2 (20ng/ml)	✓	✓	✓	✓	✓
EGF (20ng/ml)		✓	✓	✓	
SCF (25ng/ml)					✓
Flt3-L (25ng/ml)					✓
B27 (1∶50)				✓	
N2 (1∶100)				✓	
L-Glut (1∶100)		✓	✓	✓	
P/S (1∶100)	✓	✓	✓	✓	✓
BSA (10mcg/ml)				✓	
Heparin Sulfate (10mcg/ml)	✓	✓	✓	✓	✓
Substrate					
Laminin				✓	
Matrigel	✓	✓	✓		✓

### Immunocytochemistry

Cell suspensions from the negative fraction were used to make cell smears for immunocytochemistry. After smearing, cells were fixed with 4% paraformaldehyde for 10 minutes, washed twice with PBS, and air-dried. After incubation with the blocking buffer cells were stained with the following primary antibodies to surface antigens: mouse anti-SSEA-4 (Invitrogen Cat: 41–4000; 1∶100), mouse antihuman CD133 (Miltenyi Biotec, cat: 130-190-422; 1∶10) and mouse anti-human CD34 (abcam Cat:ab6330; 1∶100) with cells previously blocked. Bound antibodies were detected using secondary donkey anti-mouse antibody conjugated with Alexa Flour 568 (Invitrogen Cat: 989784). Negative controls were incubated only with the secondary antibody. Nuclei were counterstained with Hoechst 33258 (Sigma-Aldrich). All samples were viewed and imaged using Axiovert 135 fluorescent microscope with a C14 digital camera (Jenoptik). Image collection and analysis was performed using Openlab, Volocity (Improvision) and ImageJ 1.44o software [18].

### Statistical Analysis

All data are represented as mean ± standard errors of the mean (SEM) and analyzed by unpaired t test and plotted using Prism version 5.0 (GraphPad Software, Inc.); p values <0.05 was taken to be significant. A minimum number of 4 samples were used for each experiment.

## Results

### Recovery of the Lin^−^CD45^−^ Fraction from Cord Blood Total Nucleated Cells (TNCs) using either Lysis or Ficoll

**Figure 1:**
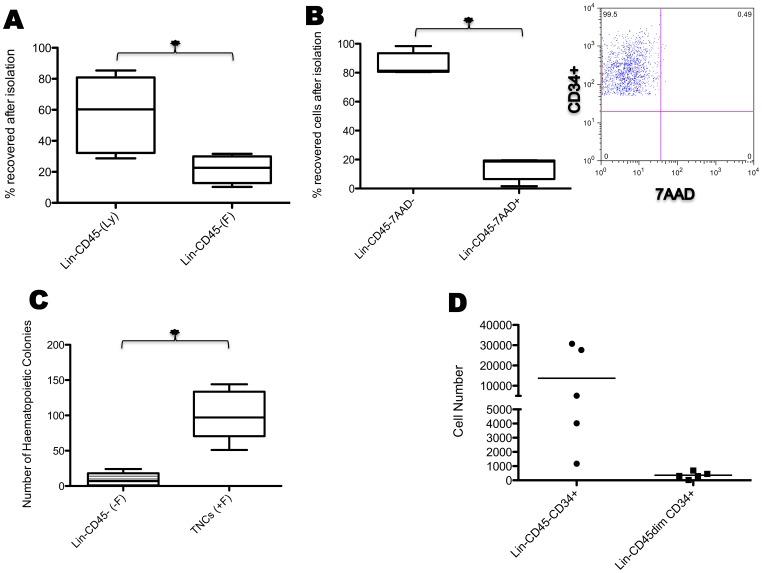
Recovery, viability, and clonogenic potential of the Lin^−^CD45^−^ population. (A) The box plots show the percentage of cells recovered after magnetic isolation using lysing buffer (Ly) or Ficoll gradient centrifugation (F) (*p = 0.0306). (**B**) The box plots show the percentage of Lin^−^CD45^−^ CXCR4^+^/CD34^+^/Nestin^+^ viable (7AAD negative) and dead (7AAD positive) cells using lysing buffer: note that the percentage of viable cells is significantly higher than that of dead cells (n = 5; *p = 0.0001); and the gate shows the 7AAD negative Lin^−^CD45^−^CD34^+^ as an example (Staining for 7AAD and Nestin is not technically possible as Nestin is an intracellular staining). (**C**) The box plots show the number of colonies formed by cells isolated using lysis buffer after 14 days in culture (n = 5; *p = 0.0005). (**D**) Absolute count of both Lin^−^CD45^−^CD34^+^ and Lin^−^CD45dimCD34^+^ cells plated in CFUs. *CFUs = Colony Forming Units. (–F) = negative fraction. (+F) = positive fraction.*

We assessed whether recovery of the Lin^−^CD45^−^ fraction differed when lysing buffer or Ficoll density centrifugation were used to prepare TNCs. As shown in Fig. 1A, the percentage of Lin^−^CD45^−^ cells recovered was significantly lower (p = 0.0025) after cell isolation with Ficoll (21.79±4.475 N = 4) than with lysis buffer (60.42±6.044 N = 10). With both procedures the main contaminants consisted of platelets, other mature cells, and to, a minimal extent, of Lin^−^CD45*dim*CD34^+^ cells. To assess the viability of cells (on day 0) prepared with lysis buffer, 7AAD staining followed by flow cytometry analysis was performed. More than 80% of cells within the Lin^−^CD45^−^ population were negative for 7AAD (85.90±3.453 N = 5) indicating that the majority of the cells were not apoptotic/necrotic (Fig. 1 B).

### Immunophenotypic analysis of TNCs

**Figure 2:**
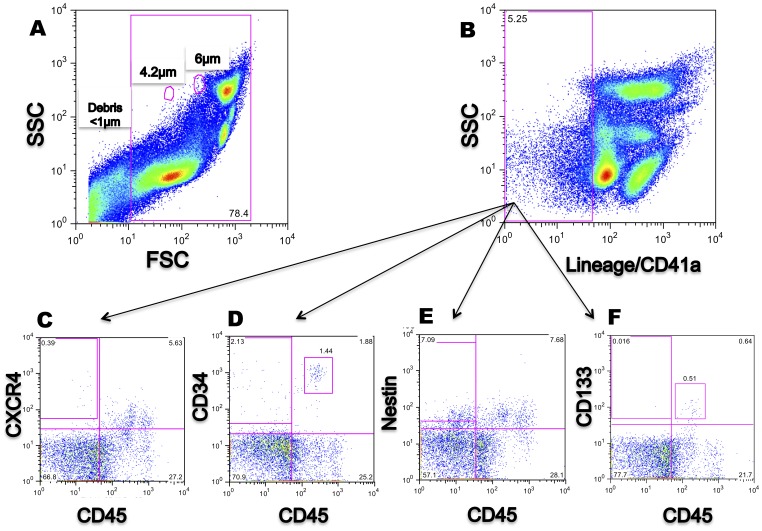
Characterization of cord blood mononuclear cells (CBMCs) isolated using the lysis protocol. (**A**) Debris is excluded from the whole CBMC in an open scale using beads as a size marker (4.2 µm and 6 µm). (**B**) Gate set to exclude Lin^+^/CD41a^+^ cells. (**C)** CXCR4^+^ is detected in the Lin^−^CD45^−^ fraction. (**D**) CD34^+^ is detected in the Lin^−^CD45^−^ and Lin^−^CD45*dim* fractions. (**E**) Nestin is detected in the Lin^−^CD45^−^ fraction. (**F**) Lin^−^CD45*dim*CD133^+^ is detected but CD133^+^ is not detected in the Lin^−^CD45^−^. Events analysed: >100,000.

We characterised the antigenic phenotype of the TNCs by flow cytometry focusing on the Lin^−^CD45^−^ cell fraction. Standard flow cytometry-based protocols for HSC usually exclude events smaller than 6 µm as this fraction is mainly composed of erythrocytes, platelets, and cellular debris [19], [20]. As previous reports suggested that Lin^−^CD45^−^ cells are smaller than HSC, with a size between 2–7 µm [3], [4], [5], we applied a log scale to the Forward scatter to including events smaller than 6 µm using beads as size markers. When events starting from 3 µm were included (Figure 2A), cells positive for Lin and CD41a, a specific platelet marker, were excluded by gating (Figure 2B); expression of CD45, CD133, CD34, CXCR4 and Nestin was assessed in the Lin^−^ gate separately in samples (Fig. 2C–F). The Lin^−^CD45^−^ population expressed CD34, CXCR4, and Nestin, but not CD133.

### Characterisation of the Lin^−^CD45^−^ Cell Fraction

The Lin^−^CD45^−^ population was further characterized by flow cytometry, immunocytochemistry and RT-PCR.

**Figure 3:**
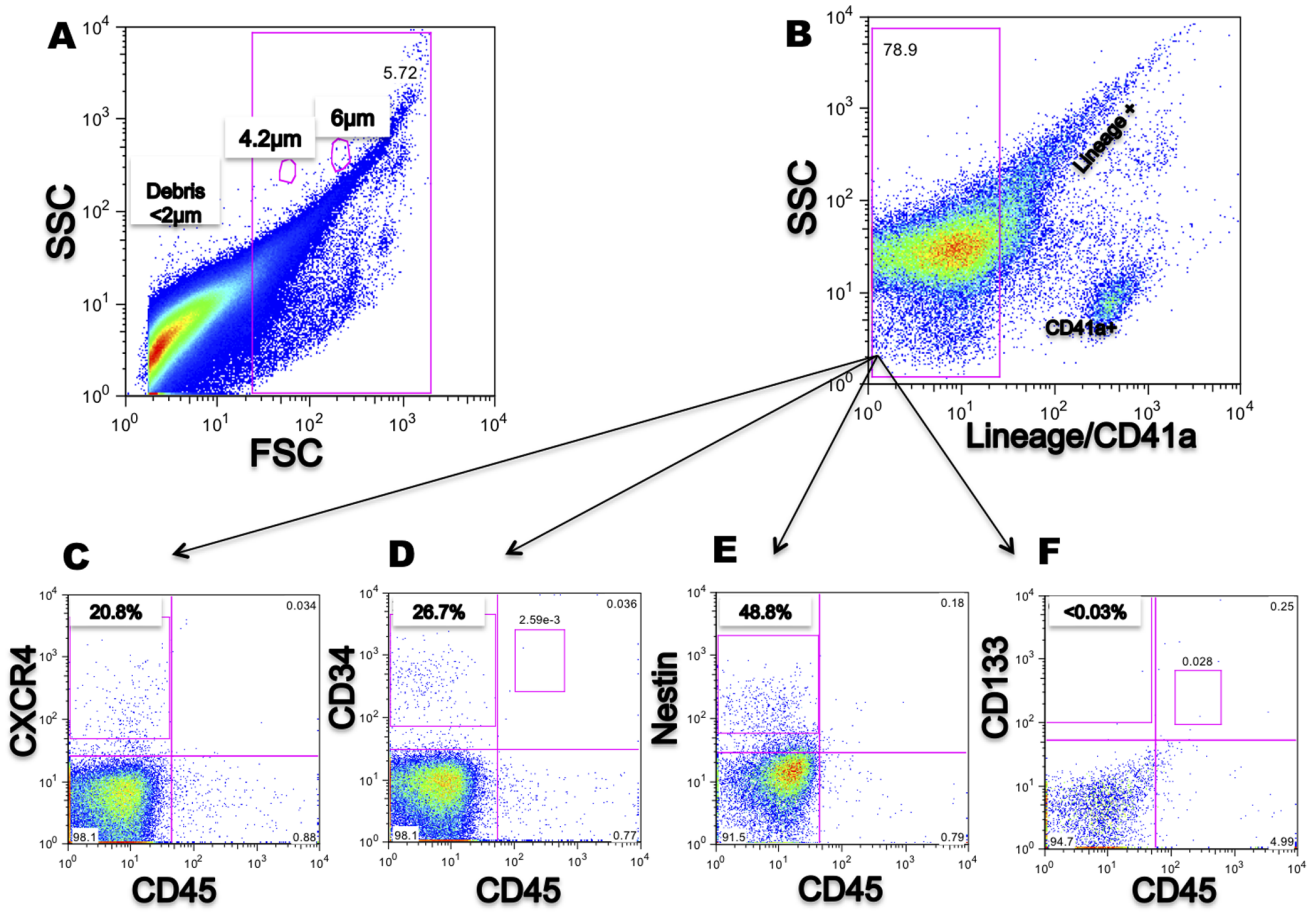
Characterisation of the Lin^−^CD45^−^ population after isolation with magnetic columns. (**A**) Debris (<1 µm) is excluded from the negative fraction. (**B**) Gate shows the exclusion of Lin^+^/CD41a^+^ cells as main contaminants and the gating of Lin^−^CD45^−^ population. **(C**, **D**, **E)** CD34^+^, CXCR4^+^ and Nestin^+^ are detected in the Lin^−^CD45^−^ fraction. (**F**) CD133^+^ is not detected in the Lin^−^CD45^−^fraction. (**C, D, E,** and **F** percentages represent the mean from 4 different samples). Events analysed: >100,000.

**Figure 4:**
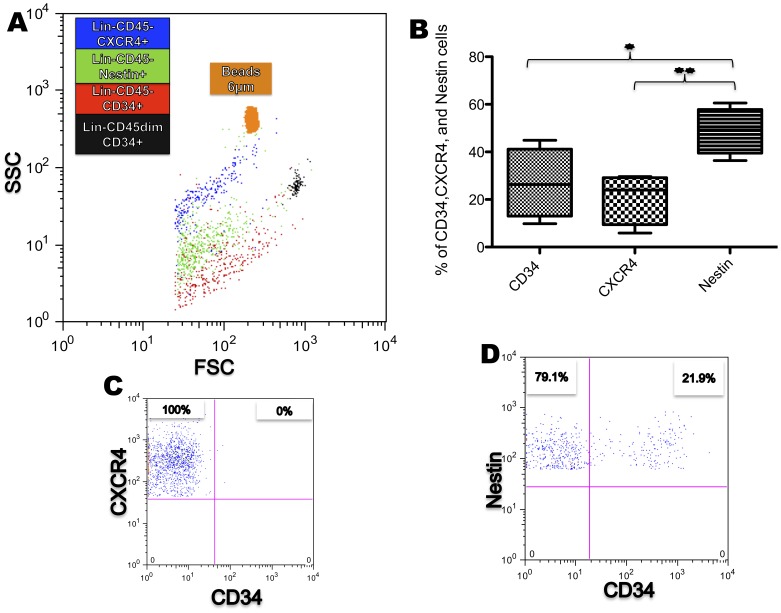
Heterogeneity of the Lin^−^CD45^−^ population. (**A**) SSC and FSC back gate show CXCR4^+^, CD34^+^, and Nestin^+^ subpopulations compared to specific size beads of 6 µm and the Lin^−^CD45dimCD34^+^ (black); they have the same range of size in FSC but are allocated differently in SSC. (**B**) The box plot shows the percentage of CD34^+^, CXCR4^+^ and Nestin^+^ cells; note that Nestin^+^ cells are the larger population within the Lin^−^CD45^−^ cell fraction. (n = 4; *p<0.05/**p<0.005). (**C**) Gate shows that CXCR4^+^ cells are negative for CD34 (**D**) Gate shows Nestin^+^ CD34^−^ and Nestin^+^ CD34^+^ cells. (**C** and **D** percentages represent the mean from 4 different samples).

Flow cytometry showed that CD34, CXCR4, and Nestin positive cells were consistently detected in all cell preparations (n = 4; Fig. 3C–E); in contrast, detection of CD133 was a rare event and most samples were negative (<0.03%, n = 4; Fig. 3F). Interestingly, Nestin+ and CD34^+^ cells were different in size from CXCR4 cells, as assessed by SSC and FSC (Fig. 4A). In double-staining experiments it was found that cells positive for CXCR4 were negative for CD34 (Fig. 4C), while approximately 21% of Nestin positive cells were also positive for CD34 (20.97±7.242 N = 4; Fig. 4D). Finally of note, a high proportion of events were either very small, on the edge of the 2 µm threshold (80%), or not stained by any antibody used; these could represent cellular debris.

The Lin^−^CD45^−^ populations were separately back-gated for SSC and FSC to compare them with the Lin^−^CD45dimCD34^+^ population using beads as size markers. The Lin^−^CD45^−^ cells were found to be smaller than Lin^−^CD45dimCD34^+^ cells by SSC and FSC (Fig. 4A).

**Figure 5:**
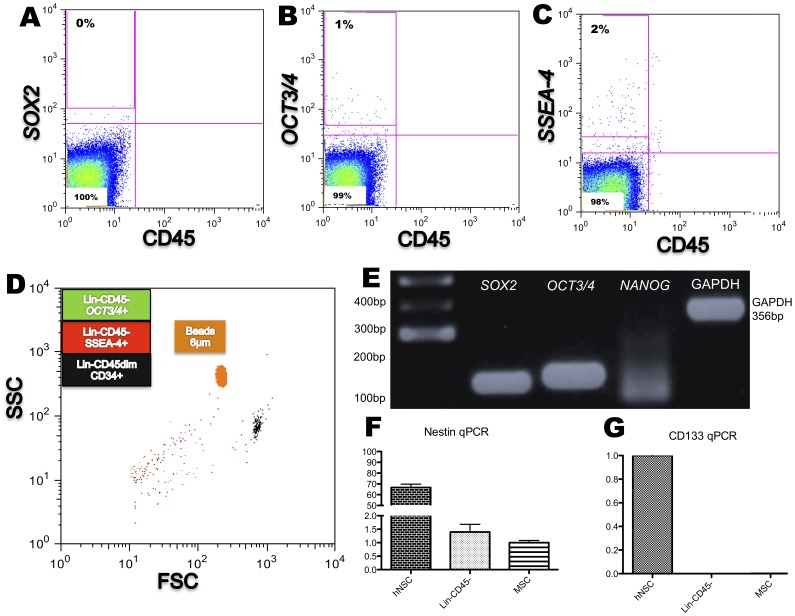
Expression of transcripts typical of pluripotent cells and CD133 in the Lin^−^CD45^−^ population. (**A–C**) Expression of the embryonic stem cell markers, SSEA-4 and OCT3/4 but not of SOX2 are detected by flow cytometry (percentage represents the mean from 5 different samples). (**D**) SSC and FSC back gate shows *SSEA-4* ^+^ and *OCT3/4* ^+^ subpopulations compared to specific size beads of 6 µm and the Lin^−^CD45dimCD34^+^ (black); they are in the same size range both in FSC or SSC. (E) *SOX2*, *OCT3/4* and *NANOG* transcripts are detected by RT-PCR. (**F and G**) Expression of Nestin and CD133 markers by qPCR in human neural (hNSC), in the Lin^−^CD45^−^ fraction, and mesenchymal (MSC) stem cells. Nestin is expressed in both Lin^−^CD45^−^ cells and MSCs cells though at a much lower level than in hNSC. Note that CD133 mRNA is not detected in the Lin^−^CD45^−^ fraction.

Expression of the pluripotent markers, SSEA-4, Sox2, and Oct3/4, in the Lin^−^CD45^−^ fraction was investigated by using flow cytomery. SSEA-4 was expressed in 2±0.3498% (N = 5) of the cells and Oct3/4 in less than 1% (Fig. 5B–C). Sox2 was not found expressed by flow cytometry (Fig. 5A). Of note, the SSEA-4 positive cells were negative for CD34 and CD133. These positive cells, when back-gated, were similar in size (by SSC and FSC) to the Nestin^+^ cells, as shown in (figure 5D).

Expression of transcripts for the pluripotent markers, SOX2, OCT3/4, and NANOG, was assessed by RT-PCR. The Lin^−^CD45^−^ fraction expressed SOX2, OCT3/4 and weakly NANOG (Fig. 5E). Expression of the stem cell markers, CD133 and Nestin, was assessed by RT-qPCR. Consistent with the flow cytometry results, the CD133 transcript, which was highly expressed in hNSC, was undetectable in the Lin^−^CD45^−^ fraction. Nestin, however, was detected (Fig. 5F–G). Nestin expression in Lin^−^CD45^−^ cells was higher than in UC-MSC, but much lower than in hNSC.

**Figure 6:**
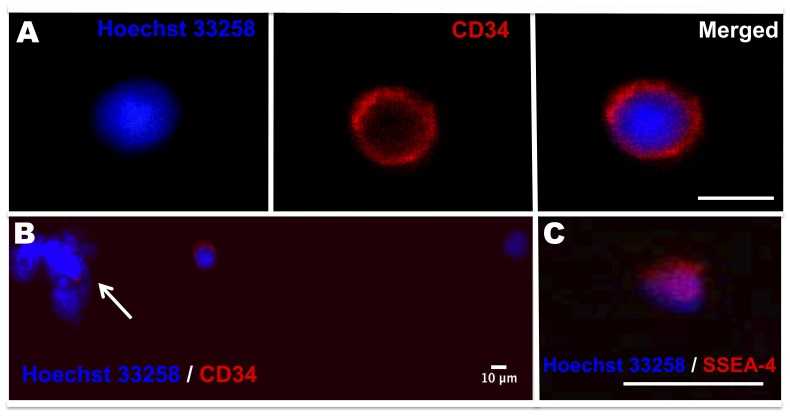
Lin^−^CD45^−^ cells show a high nuclear/cytoplasm ratio. (**A**) Immunocytochemistry shows small cells (≦10 µm) with high nuclear (blue)/cytoplasm ratio positive for CD34 (red). (**B**) Note one CD34-positive and one CD34–negative cell and an example of cell debris present in the sample (arrow). (**C**) Rare SSEA-4–positive cell. Scale bars = 10 µm (**A**–**B**) and 5 µm (**C**).

Immunocytochemistry was used to visualize the expression of CD34, CD133 and SSEA-4 in Lin^−^CD45^−^ cells (Fig. 6A–C). Staining for CXCR4 was not performed as it is also expressed in most haematopoietic cells and, therefore, its presence might be partly due contaminating cells. CD34^+^ cells were present in all the samples examined (Fig. 6A–B). No CD133^+^ cell was observed (data not shown), consistent with the flow cytometry and RT-qPCR data. Only two cells positive for SSEA-4 were detected in the 5 samples analysed (Fig. 6C). Lin^−^CD45^−^ stem cells showed high nuclear/cytoplasm ratio and a size between 6 to 10 microns (Fig. 6A–C). Cell debris, consistent with the flow cytometry results (Fig. 3), was present in cell fraction, as indicated by Hoechst nuclear staining, (Figure 6B).

### Survival and Growth of Lin^−^CD45^−^ Cells

We tested the clonogenic potential of Lin^−^CD45^−^ cells compared with the CD45^+^CD34/CD133^+^ cells present in the +F fraction using the CFU assay. The number of colonies was significantly higher in TNCs from +F (101.0±15.76 N = 5) than in Lin^−^CD45^−^ cell cultures (8.800±4.375 N = 5), p = 0.0005. Colonies originating from the Lin^−^CD45^−^ fraction could be attributed to contaminating cells with a Lin^−^CD45*dim*CD34^+^ phenotype (Fig. 1C and 1D).

We then tested the ability of Lin^−^CD45^−^ cells to survive and grow in different media known to be suitable for the expansion/differentiation of embryonic-like stem cells [3], [21], HUCBSC [17], [22], and hNSC [14] and on different substrates (Table 1). Proliferation was not observed under any of the culture conditions tested (A–E; Table 1). In culture conditions A, B, and C all cells were dead by 15 days in culture, whereas viable remaining cells were still present under condition D, a medium that supports expansion of neural stem cells and E, a medium that supports expansion of human haematopoietic cells. The surviving cells in these cultures were characterized at 2–3 weeks in culture by flow cytometry (N = 3). As summarized in Table 2 different expression profiles were observed in these cultures, with culture condition E containing a higher percentage of CD34-, CD133- and CD45-positive cells.

**Table 2:** Summary of Lin^−^CD45^−^ stem cell markers found on cells present after 2 weeks in the culture conditions shown.

Marker	Culture condition D*	Culture condition E*
**SSEA-4**	7.94% ±1.52	6.34% ±0.7543
**CD34**	1.35% ±0.4572	4.65% ±0.9699
**CD133**	1.19% ±0.3960	10.04% ±2.452
**CD45**	1.85% ±0.6015	12.42% ±1.774

Markers were assessed by flow cytometry and given as percentage of positive cells; *n = 3.

## Discussion

We have shown here that the CD45 negative and haematopoietic lineage marker negative hUCB population is heterogeneous (Table 3) and includes a Nestin^+^ subpopulation not previously described.

**Table 3:** Summary of cell populations with “embryonic-like stem cell” features reported in the hUCB Lin^−^CD45^−^ fraction**.**

Name	Immunophenotype andtranscripts	Isolation	Morphology	Survivaland Growth	Specie(s)/Tissue	PossibleFunction	Reference
**hUCB Lin** ^−^ **CD45** ^−^ **population (non-HSC)**	Lin^−^CD45^−^CD34^+^, Lin^−^CD45^−^CXCR4^+^,Lin^−^CD45^−^Nestin^+^, *SSEA-4, SOX2, OCT4, NANOG, Hoescht +.*	Lysis, Magnetic Columns.	6–10 microns, Highnuclear/cytoplasmic ratio	–	Human Cord Blood.	Quiescent.	This study
**Very Small Embryonic-like** **stem cells (VSELs)**	CD34, CD133, CXCR4, SSEA-4, SOX2,OCT4, NANOG, CD31, Hoescht(low/−/+).	Lysis, Magnetic Columns, FACS Sorting	3–7 microns, High nuclear/cytoplasmic ratio	−/+	Human Cord Blood.	Quiescent, Long-termrepopulation.	[4], [5], [23], [32]
**Cord-blood-derived** **embryonic-like stem cells (CBEs)**	SSEA-4, SOX2, OCT4, NANOG.	Ficoll density, Magnetic Selection.	3–6 microns	+	Human Cord Blood	Not reported	[3], [7], [21]

The Lin^−^CD45^−^fraction we isolated expresses several stem cell markers. Whereas we found a subpopulation expressing CD34 within this fraction, CD133 expression could not be detect either by flow cytometry, RT-qPCR or immunocytochemistry. However, when Lin^−^CD45^−^cells were cultured for 2–3 weeks, a small CD133 remaining population was detected which could possibly have been attributed to the contaminants with Lin^−^CD45*dim*CD133^+^ cells. Whether the difference between our and other author findings is due to different handling of the cells, or differences between murine and human Lin^−^CD45^−^ populations is currently unclear. Lin^−^CD45^−^ cells expressing the stem cell markers CD34 and CD133, and the chemokine receptor CXCR4 were initially described in murine bone marrow [6]. Subsequently, Lin^−^CD45^−^ cells have been isolated by a number of different groups from several animal and human sources including bone marrow [6], cord blood [3], [4], [5], [23], peripheral blood after severe damage, such as stroke [24], and mammalian ovary [25].

Several groups have reported the presence of pluripotent markers in this Lin^−^CD45^−^ stem cell population. Our results show the expression of SOX2, OCT4, and NANOG using RT-PCR, which are similar to reports by Zuba-Surma, et al. and Bhartiya, et al. [4], [5]. However, the expression of the surface SSEA-4 marker using flow cytometry was found in only a low percentage (2%) of cells in contrast to the results reported by Ali et al. [26] using a very similar isolation method. Nevertheless, the same group has also reported the expression of pluripotent markers by using RT-PCR.

Our Lin^−^CD45^−^ isolated fraction was composed of a heterogeneous population expressing different stem cell markers (CD34, CXCR4, and Nestin). With the exception of CD133, our analysis showed that the Lin^−^CD45^−^ population consistently expressed a phenotype associated with stem cells. However, this is not a homogenous population; the CD34^+^ and CXCR4^+^ populations are different to each other and do not share mutual immunophenotype. Added to these differences, our flow cytometric analysis has shown a third, Nestin^+^, population that is also negative for CXCR4. Aproximately 20% of the Nestin positive cells were also positive for CD34. A CD45^−^Nestin^+^ population has been described in the bone marrow by Mendez-Ferrer, et al. [27] and could correspond to that described by Sauerzweig, et al. [9] as small-sized nestin-positive bone marrow stem cell (SD-BMSC). The presence of mesenchymal progenitors could account for the presence of pluripotency markers whose expression has been reported in mesenchymal stem cells [16]. However, more studies are needed to clarify the relation between them.

The Lin^−^CD45^−^ fraction we isolated consists of a population of small cells with a high nuclear/cytoplasmic ratio. Our observations under the fluorescent microscope showed the cells having a high nuclear/cytoplasmic ratio but their size was slightly bigger than determined by flow cytometry (6–10 µm*)*. This is consistent with some previous findings [3], [23], [28]. However, unlike in some studies, where this fraction did not seem to incorporate the DNA stain Hoechst [5], [23], [25], in the study shown here, all nuclei of the cells isolated were Hoechst-positive. Bhartiya et al suggested that the lack of Hoechst labelling in quiescent cells was a consequence of these cells containing euchromatin. However, this explanation to explain this phenomenon still needs to be investigated as other groups have described the labelling by Hoechst in quiescent populations using immunohistochemistry [29]. Storms, et al. [30] reported a Hoechst dye negative population of high pluripotency and used flow cytometric sorting to select a small quiescent population (which they named “side population”) from bone marrow and cord blood. It will have to be established whether differences in Hoechst binding to the DNA of Lin^−^CD45^−^ reflects a true difference between the populations isolated in different laboratories, or is due to differences in the handling of the cells.

Recently, Danova-Alt et al. have reported a Lin^−^CD45^−^CXCR4^+^ population from hUCB that lacks stem cell characteristics and displays an aneuploid karyotype [23]. These results are in stark contrast to previous reports [3], [7], [21], but similar to the findings presented in this study, that provides a novel and comprehensive approach to defining the function and nature of these cells. Danova-Alt et al. however, have mostly focused on the Lin^−^CD45^−^CXCR4^+^/CD34^+^ populations, and neglected the Hoechst negative population within the flow cytometry sorted cells previously described by other groups [6], [12], [30]. In our study, haematopoietic stem and mature cells were excluded using an anti-biotin selection antibodies. Using this approach a population of small Lin^−^CD45^−^ cells with the lower possible number of haematopoietic contaminants could be isolated, with the main contaminant being platelets. These events are normally excluded using flow cytometry-based protocols for HSC [4], [19], [20]. Therefore, during our analysis a gating strategy based on that proposed by Zuba-Surma et al. [4] was employed. This resulted in a similar population as reported by Zuba-Surma et al. with the exception that CD133^+^ cells were undetectable. This is, however, consistent with the report by Danova-Alt et al. [23].

Previous studies have classified these cells based on their phenotypical properties, including “embryonic-like stem cells” [4], [5], [7], [23]. Here we highlight the difficulties in expanding the Lin^−^CD45^−^ stem cell population. These results are not unprecedented, as other groups have also reported similar problems with the expansion of this population [3], [4]. Our study has shown that even using different conditions we were not able to expand this fraction at all. In fact, the presence of a few cells that were able to expand in the Methylcellulose medium with recombinant cytokines and of a few cells surviving long-term in StemSpan® SFEM could be attributed to the presence of contaminants such as Lin^−^CD45*dim*CD34/133^+^cells. This result contrasts with most of the previous reports but is consistent with the findings of Danova-Alt et al. [23]. The presence and origin of pluripotent markers, as well as the functional role of these cells, should be investigated before attempting to classify them as embryonic-like cells. Currently, the definition of non-Haematopoietic stem cells (non-HSC) seems prudent until further studies elucidate their origin, function and possible use in regenerative medicine.

Despite of the fact that more than 80% of Lin^−^CD45^−^ cells were 7AAD negative, it was not possible to determine whether these cells were in an early apoptotic stage. Lin^−^CD45^−^ cells have been reported to bind Annexin V following red blood cell lysis, though they do not subsequently undergo apoptosis [31]. Nonetheless, the presence of haematopoietic colonies in CFUs either in –F or +F and of surviving cells in culture condition E could be taken as a rough indicator that cell viability was not severely affected by lysis.

Human umbilical cord blood has both HSC and non-HSC as several groups have demonstrated before [4], [5], [7], [23], [32]. We have shown here that the presence of a heterogeneous population lacking the expression of LCA and other mature haematopoietic markers, but expressing stem cell markers such as CD34 and CXCR4, and the tissue-committed stem cell marker Nestin, could explain the discrepancies in the literature. These differences could be due to the fact that diverse methods of isolation lead to the sorting of different populations.

In conclusion, our work has described three different cell subpopulations including Lin^−^CD45^−^ Nestin^+^, which has not been previously reported (Table 3) by any of the groups working with hUCB-derived “embryonic-like cells” [4], [5], [7], [23], [32]. However more research will be needed to establish the relationship between these populations and those previously described. A better understanding of the origin and function of this heterogeneous Lin^−^CD45^−^ stem cell population (non-HSC) is required before considering their potential use in regenerative medicine applications.

## Supporting Information

Methods S1 **Protocol for the removal of red blood cells using lysis buffer.**
(DOC)
