# Supplementary material for: Cord Blood Lin−CD45− Embryonic-Like Stem Cells Are a Heterogeneous Population That Lack Self-Renewal Capacity
Source: PLoS One. 2013 Jun 28;8(6):e67968. doi: 10.1371/journal.pone.0067968 (PMC3695943; doi:10.1371/journal.pone.0067968)
Supplement: Methods S1 — Protocol for the removal of red blood cells using lysis buffer. (DOC) [file pone.0067968.s001.doc]

**Supplementary Material and Methods.**

**Protocol for the removal of red blood cells using lysis buffer**

- Divide the blood from a cord in 20 ml aliquots/50 ml Falcon tube.
- Centrifuge at 1000g for 10 minutes at room temperature (RT).
- Remove the serum supernatant.
- Fill each tube with lysis buffer (1:5 dilution; BD Pharm Lyse, Cat:555899) and incubate for 15 minutes at RT.
- Centrifuge at 1000g for 10 minutes at RT.
- Discard the supernatant.
- Add 1-2 ml of phosphate buffered saline (PBS) to re-suspend the pellets and combine the samples in a 50 ml Falcon tube.
- Fill the tube with PBS (50ml) for washing.
- Centrifuge at 1000g for10 minutes at RT and remove the supernatant
- Do a second washing.
- Discard the supernatant and resuspend the pellet in the desired volume of PBS for cell counting.
